# Knowledge of First Aid Measures in Dental Trauma: A Survey of Teachers in the Province of Seville, Spain

**DOI:** 10.3390/children9081225

**Published:** 2022-08-13

**Authors:** David Ribas Perez, Rosmery Olivera, Asuncion Mendoza Mendoza, Beatriz Solano Mendoza

**Affiliations:** Faculty of Dentistry, University of Seville, 41001 Seville, Spain

**Keywords:** knowledge, dental trauma, school teachers

## Abstract

The high incidence of childhood dental trauma requires childcare professionals to have basic notions of dental first aid. The aim of this study is to assess the level of knowledge and aptitude (defined as the ability to operate competently in a given activity) of early childhood, primary, and/or secondary education teachers from Seville (Spain) referred to first aid measures in dental trauma. A descriptive study was conducted. The study sample consisted of 442 teachers (334 women and 108 men) coinciding proportionately with the study target population in terms of gender distribution, type of center, and teaching level. A specifically designed questionnaire was used. Most of the participants (88.5%, *n* = 391) exhibited poor knowledge and aptitude, 11.5% (*n* = 51) showed a medium level of knowledge and aptitude, and none were categorized as having a high knowledge and aptitude. As a conclusion, teachers in the province of Seville (Spain) showed marked deficiencies in their level of knowledge and aptitude referred to the adoption of first aid measures in dental trauma among their pupils.

## 1. Introduction

We define pediatric dental traumas (PDTs) as those traumas that occur to the teeth in infancy and childhood, which is when this type of damage occurs most and when it can have the greatest consequences [1].

The epidemiological data show the incidence of pediatric dental trauma to be very high in dental practice [2]. The incidence of traumatic dental injuries is 1–3%, and the prevalence is steady at 20–30% [3]. Rapid and precise management is required in such situations, and close monitoring of the lesion is, moreover, required due to the possibility of early or late complications [4]. These indications are simple to follow in adult dental trauma, but prove more complex in the case of PDTs since such cases depend upon the intervention and knowledge of the patient caregiver at the time of the accident. Pediatric dental trauma has a number of consequences. For example, in the case of tooth avulsion, an early and appropriate intervention could save the child’s tooth. Even when such injuries affect the temporary dentition, they may cause functional problems, affect the permanent tooth germ, produce infections, and cause aesthetic problems with psychosocial consequences for the child. The degree of development of the permanent teeth in children is highly variable. Depending on the age, type of tooth, moment of development of the tooth’s root at the time of the trauma, and the type of damage to the tooth in the trauma, the tooth will have a completely different treatment. For example, in a complicated fracture in a tooth with an open apex, the treatment would be an apexification. [5,6,7]. All individuals in charge of childcare, particularly professionals, sports instructors, and relatives, should be trained in what to do in situations of PDTs. How to act at the site of the accident and when and where to go should be clear concepts with an established order and they should be applied correctly. It is crucial to invest in educational programs destined to ensure coherent action since a few simple steps can improve the prognosis of dental traumas and may contribute enormously to the quality of our healthcare system [8,9].

In certain countries, knowledge on the part of the general population regarding the origin of caries is acceptable, and there are studies indicating that an adult could mention at least three actions for preventing caries [10]. International efforts in the form of global campaigns, as well as educational policies targeted to adults and children, are producing benefits [11]. Awareness-enhancing campaigns designed to promote tooth brushing are common in childhood centers. An example is the oral hygiene program for 10-year-old children in Greek schools with brushing programs and the use of fluoride toothpaste [12]. In our setting (Andalusia, Spain), early childhood and primary and secondary education teachers have received training in this field and in other aspects, such as fluoridation, thanks to the oral health promotion program “Aprende a Sonreír” (“Learn to Smile”) [13], which the regional health authorities have implemented since 2002. However, when it comes to dental trauma, these teachers evidence training shortcomings in many aspects, particularly regarding the provision of first aid at the site of the accident [14]. Intervention in such situations on the part of the teaching staff and other childcare professionals directly conditions the prognosis of dental trauma. Knowledge among these individuals of first aid measures in dental trauma is, therefore, necessary [7,15,16]. Their actions are of crucial relevance particularly in certain clinical situations, such as dental avulsión [15].

Teachers should be aware that dental trauma is a clinical emergency [4] and, as such, requires prompt attention from the pediatric dentist. They should be able to manage a clear and concise protocol with indications of unifying professional criteria, which are the same for all centers regardless of their public or private nature. Emphasis also must be placed on the need to prevent dental trauma through measures, such as the use of mouth guards during sports activities [17,18].

To date, no studies have been carried out in southern Spain on the knowledge of first aid in dental traumatology among education teachers at any level of teaching. We believe that it is really important for these professionals to have a good level of knowledge and competence in order to be able to deal with situations in which damage to teeth occurs as a consequence of blows or trauma, given that early diagnosis and treatment will lead to a much better prognosis for the affected structures. With this study, we want to demonstrate their level of knowledge in order to plan the training and educational needs for the group in terms of dental trauma. The fact of including teachers from different educational levels increases the possibility of covering the different treatment possibilities, from deciduous dentition to complete permanent dentition.

A survey involving a representative sample of early childhood, primary, and/or secondary education teachers from the province of Seville (Spain) was carried out with the aim to evaluate their knowledge and aptitude (defined as the ability to operate competently in a given activity) referred to first aid measures in dental trauma.

## 2. Material and Methods

A specific questionnaire was designed for our survey using Google Forms, with a posterior validation by university lecturers specializing in the study topic. The collected data were entered on MS Excel spreadsheets for subsequent analysis using the SPSS statistical package.

The study population of our survey comprised all active teachers during the school year 2019–2020, serving in public, private, and/or concerted schools in the province of Seville (Spain) and with teaching competences in early childhood, primary, and/or secondary education (including high school and basic and middle level professional training). The exclusion criteria for those interviewed included not having a teaching degree (because they were in training) or being a teacher of a higher level than required, as well as the fact of not being professionally active (retired, unemployed, etc.).

The health crisis caused by the COVID-19 pandemic made it necessary to administer the questionnaire online.

A sample size calculation for finite populations was carried out to determine the required number of completed questionnaires. Considering a population of 22,148 teaching professionals and assuming a sample randomized sampling with a margin of error of 5%, the minimum number of questionnaires was seen to be 378 [19] (Table 1).

### 2.1. Study Questionnaire

A specifically designed anonymous and self-administered questionnaire to be completed online was used for the study. The questionnaire was grouped into four groups with a total of 25 items.

The structure of the questionnaire begins with an introduction, followed by the demographic data of the participants. The body of the questions with 4 dimensions is organized as follows: (19 items organized into four dimensions)

(a) Evaluation of the level of knowledge: four items assessing the knowledge of the participant referred to general concepts in dental trauma.

(b) Evaluation of aptitude in relation to situations of dental trauma: eight items assessing the aptitude of the participant for providing care in the case of a pupil that has suffered tooth trauma.

(c) Experience in dental trauma: two items exploring whether the participant has personally suffered dental trauma or helped someone else who has suffered dental trauma.

(d) Training in first aid measures: five items assessing whether the participant has received training in first aid measures.

We finished the questionnaire with an offer to give talks on the subject in interested educational centers.

### 2.2. Scoring of the Questionnaire

Only the first two dimensions of the set of questions were scored based on a scale of 0 to 1 points (0 points = incorrect answer and 1 = correct answer).

(a) General knowledge of dental trauma: four multiple alternative closed response items with only one correct answer.

(b) Aptitude in relation to situations of dental trauma: eight multiple alternative closed response items with only one correct answer.

Since both dimensions comprised 4 and 8 items, respectively, the possible questionnaire scores ranged from 0–12 points. In order to categorize the quantitative variable “Questionnaire score” and transform it into an ordinal qualitative variable, we created the variable “Level of knowledge and aptitude” with the definition of three levels (Low, Medium, and High).

### 2.3. Validation of the Questionnaire

A pilot questionnaire was initially designed, comprising 30 questions and with a structure slightly different from that of the current final questionnaire. The pilot questionnaire was examined by 5 experts in the field to assess its clarity and the pertinence of the questions. Their comments also contributed to define the wording and sequence of the questions in order to make the questionnaire as effective and efficient as possible.

The COVID-19 pandemic made it necessary to resort to online technologies (WhatsApp and social networks, such as Facebook and Instagram) for the distribution and application of the questionnaire since the population lockdown at the time of the study precluded contact through other means. The education and sports authorities of Andalusia provided the contact information corresponding to all the public centers in the province of Seville, thus, allowing us to send e-mails to 670 out of a total of 799 centers, excluding those that failed to meet the study requirements. Likewise, the directors of the centers were contacted by mobile phone whenever possible.

The data obtained from the questionnaire, designed using the tool Google Forms, were entered on MS Excel spreadsheets for subsequent analysis, with the exclusion of 16 questionnaires.

The qualitative data were coded and transformed into quantitative information for processing with the SPSS statistical package. A descriptive analysis was performed with the generation of frequency tables and the calculation of measures of central tendency (mean and median), the verification of normal data distribution, the generation of cross-tables, and the calculation of Cramer’s coefficient of association to determine statistically significant correlations.

### 2.4. Patient and Public Involvement

People involved in the study were randomly selected from a public list of teachers provided by the regional government. Once the study had been carried out, they were given a specific training webinar on first aid in dental traumatology where the preliminary results of the research were summarized.

## 3. Results

The total sample size consisted of 442 participants, representing 75 municipalities of the 106 found in the province of Seville. The distribution of the sample according to gender was 24.43% for males and 75.57% for females. According to age, 7.01% of the teachers were between 20 and 29 years old, 25.34% between 30 and 39 years old, 38.91% between 40 and 49 years old, and 28.74% were over 50 years old. A total of 41.85% of the sample came from the capital city of Seville.

Regarding the type of center, more than three-quarters (76.47% of the sample) worked in the public sector. The rest worked in private or subsidized centers (5.43% and 18.10%, respectively). The level of education at which the respondents worked was 43.66% primary school teachers, 29.64% secondary school teachers, and 26.70% preschool teachers.

To conclude with these data, the years of professional experience of the respondents was 40.05% of teachers with between 11 and 20 years of experience, 27.50% with less than 10 years of experience, and 15.84% with more than 20 years of professional experience.

Of the 442 participants in the survey, 88.5% yielded a score between 0–5 (out of 12 possible points), reflecting a low level of knowledge and aptitude in relation to first aid measures in dental trauma. Only 11.5% of the teachers scored between 6–9 points, corresponding to a medium level of knowledge and aptitude. None of the participants scored above 9 points (a high level of knowledge and aptitude).

The participants scored better in the first dimension (knowledge) than in the second (aptitude). Specifically, in relation to the dimension “Level of knowledge”, 52.3% obtained medium level scores and eight teachers even obtained high scores. In contrast, a full 90.7% produced low scores for the dimension “Aptitude” (Table 2).

Table 3, Table 4 and Table 5, respectively, evidence the following: Only 3.8% of the participants reported feeling able to reimplant an avulsed tooth. Only 5.4% of the participants would transport the avulsed tooth in a container with milk. Of note is the observation that 70.1% would use toilet paper or a handkerchief. Only 14% of the participants would go to the pediatric dentist, 39.8% would report to the primary care center, and 36.2% would go to the hospital emergency room.

On the other hand, as the age of the participants increased, the percentage of those showing low knowledge and aptitude scores was seen to decrease (from 93.5% to 80%). Likewise, with increasing age the percentage of participants yielding medium scores was seen to rise (from 6.5% in those under 29 years of age to 20% in those over age 60).

The recorded Cramer’s coefficient of association of over 0.6 indicated a strong and statistically significant correlation between the “Level of knowledge and aptitude” and the variable “Age” of the participant (Table 6).

With regard to the training received, almost half (44.4%) of the teachers who had received training in first aid measures in dental trauma showed a medium level of knowledge and aptitude. In contrast, this percentage dropped to only 10.9% among those teachers who had received no such training. Although Cramer’s coefficient of association indicated no statistically significant correlation between the variables, the analysis of the table appeared to indicate otherwise since the proportion of teachers with medium level scores was higher in the group that received training than in the group without training (Table 7).

## 4. Discussion

The clinical practice guides of the International Association for Dental Traumatology (IADT) confirm that the prognosis in cases of dental trauma is directly influenced by the time elapsed from injury to the start of adequate treatment [1,7,16]. There is scientific evidence and general agreement in dental traumatology that in certain clinical situations, such as tooth avulsion, correct intervention at the site of the accident has an impact upon the prognosis [15,20]. Although many countries in Europe have implemented prevention and immediate intervention campaigns with successful results [21,22], no such initiatives in oral health are found in Spain, designed to instruct and promote adequate intervention on the part of the population or teaching professionals in situations of dental trauma. 

Taking into account that certain age groups (particularly the pediatric population) are more likely to suffer such injuries, it is advisable for those in charge of children (such as early childhood and primary and/or secondary education teachers) to be familiarized with a first aid intervention protocol in dental trauma. In our study, the teachers were not sufficiently trained in this area as evidenced in 97.7% of the participants in the survey. This may be explained by the fact that a full 98% of the teachers claimed to have received no information about first aid measures in dental trauma through any means.

Studies similar to our own report somewhat more positive results in this regard, with an incidence of subjects able to deal with pediatric dental traumas (PDTs) of under 25% (range 12–23.5%) [23,24,25,26,27]. Possibly, as a consequence of the absence of educational programs for teachers, we have obtained these results. This explains why 88.5% of the participants in our survey presented a deficient level of knowledge and skills in relation to first aid measures for dental traumas.

A total of 15.6% of the participants expressed an interest in receiving training in this area. This low figure may be due to the fact that school campaigns usually focus on the prevention of caries and to the widespread lack of knowledge of the frequency and sequelae of dental traumatisms.

In contrast to our results, other similar studies have reported a wish to receive more information about PDTs on the part of most teachers [23]. One of the factors that typically influences knowledge and aptitude in relation to certain subjects is gender. However, gender was not found to be relevant in our case since very similar percentages were recorded for both men and women as follows: 89.8% and 10.2% of the women showed low and medium scores, respectively, versus 84.3% and 15.7% of the men.

The differences between the two genders were not statistically significant. The teaching staff requirements vary depending on the type of center. However, our results show that regardless of the type of center (public, private, or concerted), none have dedicated resources to ensure that their teaching staff receive basic training in this area. We observed no statistically significant relationship between the knowledge or aptitude of the teachers and the public or private nature of the center.

In contrast, a statistically significant relationship was observed between the level of knowledge and aptitude and the fact of having received training in dental first aid measures. Of the nine teachers (2% of the sample) that had received training in this area, 44.4% (i.e., nearly half) showed a medium level of knowledge and aptitude. In comparison, out of the 433 teachers (98% of the sample) that had received no such training, only 10.9% presented a medium level of knowledge and aptitude.

The aforementioned 2% of the participants with training is much lower than in the rest of the studies found in our review of the literature. In effect, 8 studies reported first aid training in dental trauma in less than 75% of the participants [23], while 20 studies reported figures of under 50% [24,25,26,27,28,29,30,31].

In reply to the question “If the tooth is broken, can the broken part be joined again?”, only 7% were aware that joining is possible in the case of a permanent tooth. Ten studies in other countries have found that 50% of the teachers are aware of the importance of finding the broken tooth fragment [23,26,31].

In reply to the question “If you find the permanent tooth, would you feel able to reimplant it?“, a total of 84.2% of the participants in our survey stated that they would not be able to. While the possession of knowledge referred to performing immediate reimplantation of a permanent tooth (recorded in five out of six studies in a meta-analysis published in 2020 [23]) or no immediate reimplantation of a temporary tooth (recorded in seven out of ten studies in the mentioned meta-analysis [23]) is important, it is also necessary to feel able to do so in a critical moment [22].

With regard to the storage and transport medium of the tooth, only 5.4% were aware that milk is the ideal medium. Only 25% of the teachers in other studies [26,27,28,29,30,31] were aware of this fact or cited the saliva of the affected child as the indicated medium. Of note is the observation that 70.1% of the participants in our survey would use toilet paper or a handkerchief, which would cause the death of the periodontal ligament cells and, thus, result in irreversible damage. In the present study, only 15% of the participants would seek specialized help from a pediatric dentist.

In contrast, in our review of the literature, over 70% of the teachers would report to a doctor or a specialist for adequate management [20,23]. These data evidence a clear lack of preparation in these aspects compared with other countries, despite the fact that the required knowledge is easy to assimilate for teaching professionals. Only 3.8% would reimplant the tooth, despite the fact that viability of the latter increases if the period of dryness outside the mouth is less than 20 min. This means that viability could be facilitated provided reimplantation is made at the site of the accident.

A total of 10 out of 17 studies conducted in other geographical settings have reported knowledge of the importance of the time of reimplantation [23].

The time factor is crucial in dental trauma, and, in this regard, 54.1% of the participants considered it desirable to seek help in under 30 min. The mistake of reporting first to a primary care center (39.8% of the participants in the survey) or to the hospital emergency room (36.2%) rather than to a dentist (6.1%) or, more specifically, to a pediatric dentist (14%) could be avoided through adequate training on how to deal with a dental emergency. With acquisition of the required knowledge, the observed figures could be expected to improve considerably since they are a consequence of a serious lack of information. In any case, we found that with advancing age, the incidence of a low level of knowledge and aptitude decreased among the teachers (from 93.5% to 80%), while the incidence of a moderate level of knowledge and aptitude increased (from 6.5% in those under 29 years of age to 20% in those over the age of 60), although Cramer’s coefficient of association did not find this correlation to be statistically significant. The present study is limited to the province of Seville (Spain), but this is not a local problem and other studies have reported very similar data.

In a study conducted in 2009 in Brazil, only 2.2% of the teachers were found to have good levels of knowledge [31]. In turn, another study carried out in the United States found that 44% of the surveyed teachers did not feel able to reimplant a tooth and that 28% did not know how to do so [22].

A European study in 2005 found that 74.3% of the surveyed teachers admitted having no knowledge on dental traumas [21]. Lastly, a study carried out in the United Kingdom in 2001 found that 66.1% of the study subjects had received no information or advice about dealing with dental traumas [20].

Finally, one of the limitations of the study is that it has been carried out in the province of Seville and we suppose that the results could be extended to the rest of the region of Andalusia in southern Spain, for which we would need a much larger sample of teachers to be able to extrapolate the results.

## 5. Conclusions

The early childhood and primary and/or secondary education teachers in the province of Seville (Spain) present marked deficiencies in their level of knowledge and aptitude referred to the adoption of first aid measures in dental trauma among their pupils. Institutional campaigns are needed to improve the level of knowledge in this field among the teachers of the province.

## Figures and Tables

**Table 1:** Comparative distribution of the study population and sample in terms of gender, type of center, and level of teaching.

		Population	Sample
**Gender**	Men	27.47%	24.40%
	Women	72.53%	75.60%
**Type of Center**	Public	69.28%	76.50%
	Private	30.72%	5.40%
	Concerted		18.10%
**Level of teaching**	Early Choldhood	25.75%	26.70%
	Primary	37.26%	43.70%
	Secondary	36.99%	29.60%

**Table 2:** Level of knowledge versus level of aptitude in relation to first aid measures in dental trauma.

Level of Knowledge of the Early Childhood, Primary and/or Secondary Education Teachers on First Aid in Dental Traumatology						Level of Aptitude of the Early Childhood, Primary and/or Secondary Education Teachers on First Aid in Dental Traumatology					
	Fr.	%		Fr.	%		Fr.	%		Fr.	%
0	82	18.6	Low (0–1)	203	45,9	0	42	9.5	Low (0–3)	401	**90.7**
1	121	27.4				1	121	27.4			
2	159	36	Medium (2–3)	231	**52.3**	2	144	32.6			
3	72	16.3				3	94	21.3			
4	8	**1.8**	High (4)	8	1.8	4	32	7.2	Medium (4–6)	41	9.3
Total	442	100		442	100	5	6	1.4			
						6	3	0.7			
						7	0	0	High (7–8)	0	0
						8	0	0			
						Total	442	100		442	100

**Table 3:** Tooth avulsion reimplantation.

In Case You Find the Permanent Tooth, Do You Feel Able to Reimplant It?		
	Frequency	Percentage
I would look for health personnel from the school to do it	32	7.2
Don’t know/No answer	14	3.2
No, never	372	**84.2**
**Yes**	17	**3.8**
Only sometimes	7	1.6
Total	442	100

**Table 4:** Storage medium and transport.

If You Have the Tooth, How Would You Transport It?		
	Frequency	Percentage
In the child’s mouth	11	2.5
In toilet paper or a clean handkerchief	310	**70.1**
In a container of water	20	4,5
**In a container with milk**	24	**5.4**
In a container with saline solution	77	17.4
Total	442	100

**Table 5:** Where to seek professional help.

If a Student Had a Tooth Broken or Knocked Out Because of a Severe Blow to the Mouth, Where Is the First Place You Would Seek Help?		
	Frequency	Percentage
Health Center or Primary Care	176	39.8
Dentist	27	6.1
**Paediatric Dentist**	62	14
Don’t Know/No Answer	17	3.8
Emergency Room	160	36.2
Total	442	100

**Table 6:** Level of knowledge and aptitude according to age.

			Age					Total
			20–29 Years	30–39 Years	40–49 Years	50–59 Years	60–65 Years	
**Level of Knowledge and Skills**	**Low**	Count	29	101	151	94	16	391
		% within age	**93.50%**	90.20%	87.80%	87.90%	**80.00%**	88.50%
	**Medium**	Count	2	11	21	13	4	51
		% within age	**6.50%**	9.80%	12.20%	12.10%	**20.00%**	11.50%
**Total**		Count	31	112	172	107	20	442
		% within age	100.00%	100.00%	100.00%	100.00%	100.00%	100.00%

V de Cramer = 0.622.

**Table 7:** Level of knowledge and aptitude according to training received in relation to first aid measures in dental trauma.

			Training First Aid		Total
			No	Sí	
**Level of Knowledge and Aptitude**	**Low**	Count	386	5	391
		%	89.10%	**55.60%**	88.50%
	**Medium**	Count	47	4	51
		%	**10.90%**	**44.40%**	11.50%
Total		Count	433	9	442
		%	100.00%	100.00%	100.00%
**V de Cramer =**	**0.002**				

## Data Availability

The study did not report any data.
